# An Investigation on Bilateral Asymmetry in Electrodermal Activity

**DOI:** 10.3389/fnbeh.2019.00088

**Published:** 2019-05-07

**Authors:** Andreas Bjørhei, Filip T. Pedersen, Saeed Muhammad, Christian Tronstad, Håvard Kalvøy, Slawomir Wojniusz, Oliver Pabst, Stefan Sütterlin

**Affiliations:** ^1^Department of Mechanical, Electronic and Chemical Engineering, Oslo Metropolitan University, Oslo, Norway; ^2^Department of Clinical and Biomedical Engineering, Oslo University Hospital, Oslo, Norway; ^3^Department of Physiotherapy, Faculty of Health Sciences, Oslo Metropolitan University, Oslo, Norway; ^4^Division of Clinical Neuroscience, Oslo University Hospital, Oslo, Norway; ^5^Department of Physics, University of Oslo, Oslo, Norway; ^6^Faculty of Health and Welfare Sciences, Østfold University College, Halden, Norway

**Keywords:** electrodermal activity, emotional arousal, sympathetic nervous system, skin conductance, bilateral asymmetry

## Abstract

The Multiple Arousal Theory (Picard et al., 2016) was proposed to explain retrospective observations of bilateral differences in electrodermal activities occurring in threat-related high-stake situations. The theory proposes different cortical and subcortical structures to be involved in the processing of various facets of emotional states. Systematic investigations of this effect are still scarce. This study tested the prediction of bilateral electrodermal effects in a controlled laboratory environment where electrodermal activity (EDA) was recorded bilaterally during normal activity and two stress-tasks in 25 healthy volunteers. A visual search stress task with a performance-related staircase algorithm was used, ensuring intersubjectively comparable stress levels across individuals. After completion of the task, a sense of ownership of an attractive price was created and loss aversion introduced to create a high-stake situation. Confirmation of the theory should satisfy the hypothesis of a bilateral difference in EDA between the dominant and non-dominant hand, which is larger during high-stake stressors than during low-stake stressors. The bilateral difference was quantified and compared statistically between the two stress-tasks, revealing no significant difference between them nor any significant difference between the stress tasks and the period of normal activity. Subgroup analysis of only the participants with maximum self-rating of their desire to win the price (*n* = 7) revealed neither any significant difference between the two tasks nor between the stress-tasks and the period of normal activity. Although the theory was not confirmed by this study, eight cases suggestive of bilateral difference within the recordings were identified and are presented. Because the study is limited in using one of several possible operationalizations of the phenomenon, it is not possible to draw a general conclusion on the theory. Nevertheless, the study might contribute to a better understanding and encourage systematic review and hypothesis development regarding this new theory. Possible explanations and suggestions for future pathways to systematically investigate the Multiple Arousal Theory are discussed.

## Introduction

Side differences in electrodermal activity (EDA) has been reported since the 1920s, and was explored by many investigators in the 1970s and 1980s, focusing on the possible laterality of excitatory and inhibitory CNS influence on EDA, and the use of EDA in measuring hemispheric specialization. A review covering this literature can be found in Hugdahl (1984) evaluating 51 papers, and the topic of electrodermal lateralization and hemispheric asymmetry is covered in Boucsein (2012) ch 3.1.4. In the recent article «Multiple arousal theory and daily-life electrodermal activity» Picard et al. (2016) report unintended findings of occasional lateral asymmetries of EDA and discuss their results in the context of a suggested “Multiple arousal theory.” In a series of case studies the long-term EDA measurements were for the most part symmetric between the right and the left side, however a strong asymmetry with markedly increased skin conductance level (SCL) on the right side was observed in what the authors interpreted later as “high-stake emotional” or “threat-related” situations (Picard et al., 2016). The authors provided a theoretical background for the multiple arousal theory on the basis of previous EDA and imaging studies relating the notion of relatively increased right-lateral EDA to right-hemispheric amygdala activation, which has previously been associated with threat-related emotional responses (Mangina and Beuzeron-Mangina, 1996; Lanteaume et al., 2007; Monk et al., 2008). The interpretation that different brain regions can cause different arousal patterns around the body, might be key to a better understanding of psychophysiological correlates of distinct emotional states, and highly interesting for new applications of wearable sensors.

The suggested “multiple arousal theory” raised considerable interest and resulted in several commentaries followed by authors’ response (Critchley and Nagai, 2015; Mendes, 2016; Norman, 2016; Picard et al., 2016; Richter, 2016; Sabatinelli, 2016). While these commentaries were in general positive toward the concept of multiple arousal theory, the need for more systematic research including testable and falsifiable predictions has been voiced (Richter, 2016). In a recent study by Kasos et al. (2018), laterality of EDA was found due to emotions induced by music, in particular for palmar EDA during states of fear and sadness, supporting the Multiple Arousal Theory. The main objective of this study was to investigate whether EDA bilateral asymmetry could be reproduced in a laboratory setting using a paradigm that resembled Picard’s “high-stake” situation.

Since scientific definitions of arousal are multiple, contradictory, and fuzzy (Mendes, 2016), we used as a starting point of this study the retrospectively developed interpretation suggested by Picard et al. (2016). We interpreted Picard’s term «high-stake» as corresponding to the psychological term «salience» and interpreted the term «threat» as resembling an intensively negative, possible, but yet uncertain anticipated future event. To realize such a situation with a realistic risk of a negative experience, we induced a loss aversion situation (Tversky and Kahneman, 1974, 1981). The concept of loss aversion is a well established and robust phenomenon known to require effortful self-regulation, prefrontal inhibitory activation, limbic inhibition and has been robustly shown to predict economic and social decisions in high-stake situations in economics and decision theory (De Martino et al., 2006; Tom et al., 2007; Sokol-Hessner et al., 2012). Loss aversion can be induced by so-called “gambling tasks” in which the participant is given a sense of preliminary ownership of a valuable good (salience) that is in danger to be lost (threat). The paradigm reliably induces the urge to avoid the loss. Loss aversion scenarios have been shown to involve limbic structures associated with threat detection as well as prefrontal structures associated with emotion regulation and behavioral control (e.g., De Martino et al., 2006; Sütterlin et al., 2011). The magnitude of loss aversion depends not only on the object’s value and the perceived risk of loss, but also on the individual’s emotion regulation ability and resulting physiological arousal (Sütterlin et al., 2011; Sokol-Hessner et al., 2012). In the present study, we therefore introduced in a sample of healthy participants a sense of ownership of a subjectively valuable object (iPad) and assessed via self-report the motivational level as to which this object was desired. In a second step, participants were after completion of an exhausting visual search task informed that they fulfilled the criteria and are entitled for an iPad due to their excellent performance, but that their entitlement might be withdrawn if they could not reproduce the same performance level in an additional round.

According to prospect theory (Kahneman and Tversky, 1979), the sense of ownership acquired by the information about entitlement to win the price that was visibly placed on the table, causes a re-setting of reference points along the value axis. Prospect theory predicts that at this stage the iPad was not longer considered to be a possible win, since the win was achieved and thus a sense of ownership was acquired. Instead, the situation was associated with the potential loss of the newly acquired valuable good. Based on a large body of research on framing effects in gambling tasks, we assume that the risk of possibly losing the iPad created a loss aversion, a psychologically stressful state we interpret as equivalent to an emotionally intensive high-stake situation in the sense suggested by Picard et al. (2016).

The present study aimed to determine whether there is bilateral asymmetry in EDA related to high-stake stressors as operationalized in a loss-aversion paradigm. Based on the initial findings proposed in the Picard et al. (2016) paper, a confirmation of the multiple arousal theory should satisfy the hypothesis of a bilateral difference in EDA parameters between the dominant and non-dominant hand, which is larger during high-stake stressors than during low-stake stressors.

## Materials and Methods

### Participants

A power analysis indicated that 20 subjects would be necessary to obtain a power of 0.95 for detecting an intraindividual change at 1/8th of the interindividual variance with an alpha of 0.05. Thus, the statistical power of our hypothesis test would be above 0.95 for detecting a small change effect of bilateral asymmetry.

Twenty-five healthy adults (mean age: 31.28, sample standard deviation: 14.06, 64% male) were recruited primarily from the student population and gave written informed consent. Exclusion criteria were age below 18, a diagnosis of either diabetes, hypo- or hyperthyroidism, nerve injury, or feeling ill on the day of the session. All participants were recruited through OsloMet – Oslo Metropolitan University. The participants were asked whether they were left or right-handed in order to sort the recordings into EDA belonging to the dominant or non-dominant hand. The participants received no compensation for participation beyond the possibility to win a price (an iPad Air model 2 worth approximately 480$).

### Material

For recording of skin AC (alternating current) conductance at several skin sites simultaneously (Tronstad et al., 2008), two Sudologgers (BioGauge AS, Oslo, Norway) were used together with solid-gel ECG electrodes (Kendall 1050NPSM Neonatal Electrodes) and a PC for recording by wireless connection to the device. Similar to the Picard et al. (2016) study, no filtering or parameterization was used on the measurement, which represents the SCL recorded over time.

In order to induce mental stress in the test subject for a short period of the experiment, the LARA task was employed (Thum et al., 1995). The task is run by a computer application which records a reaction time by testing the ability to recognize two decimal numbers in a matrix. The subjects are presented a 6 × 6 matrix of two-decimal numbers and are instructed to look for two specific numbers out of the 36 and answer whether they see one, two, or none of the specific numbers. The time to input is limited, will decrease if the answer is correct, and increase if the answer is incorrect or time runs out. In this way, the stress task will induce a subjectively identical level of mental stress regardless of participants’ interindividual perceptual or motor abilities as it continuously adjusts the time pressure according to ongoing task performance. The test runs for 4 min in total.

### Procedure

The experiment took place in a calm indoor environment with normal room temperature. The subject rested for approximately 10 min for habituation before being connected to the measurement equipment by two reference electrodes and one electrode at both hypothenar sites bilaterally. The experimental session was scheduled as shown in Table 1 and lasted roughly 2.5 h. The experiment consisted of two phases; in the first phase, the subject took part in any normal activity except physically straining. This could be activities such as going to class or reading a book. During the second phase, the subject is exposed to mental stress in order to observe changes in bilateral EDA.

**Table 1:** Typical timeline for the experiment.

Phase 1 (any typical daily, non-physical activity)				Phase 2			
	
Rest	Class	Break	Class	Stress tasks			
				
				Rest	LARA1	Rest	LARA2
10 min	45 min	15 min	45 min	5–10 min	5 min	10 min	5 min


In order to test the hypothesis on bilateral EDA asymmetry and the relation to high-stake stress, the LARA stress task was conducted two times successively with a short resting period in between. Before the first test (LARA1 in Table 1) the participant was instructed to solve the task as fast and precisely as possible. After completion of the first test (LARA1), the participants were told that their performance satisfied the criteria and they were now entitled to receive the price, while the price (an unopened iPad Air II 16GB WIFI, price approximately 480$) was placed on the table. Then, the subject was told that in order not to *lose* the price, the test had to be repeated for a convincing, i.e., reliable, result, by obtaining a score as similar as possible to the previous round. Literal instruction (translated from Norwegian): “This was a very good result. You now fulfill all criteria for winning the price.” – Price is physically placed on the table in good sight of the participant – “But in order to not lose the price you must repeat the test and obtain a score as equal as possible in order to convince us that your result was not occasional.”

To verify the subjective salience of the stimulus (price) and thus ensure that a high-stake situation created at LARA2 phase was indeed created, all subjects were asked to rate their degree of desire for winning the price on a scale from 1 to 10.

### Data Reduction and Analysis

For temporal comparison of skin conductance between the two hands, the recordings were synchronized based on the start time of LARA1 (set to time = 0), starting from -90 min until completion of LARA2. Within this interval, the median and 95% confidence interval over all subjects were calculated separately for the dominant and non-dominant hands, and plotted in the same graph for visual inspection. The median was used in favor of the mean due to a skewed between-subjects distribution, and the confidence intervals for the median were calculated based on the bootstrap method. Due to differences in pause lengths between the two LARA tests between the experiments, data points during this pause interval were cut out in order to synchronize the recordings with the start of LARA2.

For further inspection of bilateral asymmetry, the bilateral conductance difference was obtained by sample-wise subtraction and plotted over time within the same interval. This difference was represented as the median of the skin conductance difference (dominant minus non-dominant hand), the median of the absolute value of this difference, and the median of the percent-wise ratio between the dominant and non-dominant hand. These differences were plotted in the same way including the 95% confidence bounds of the medians.

Testing of the hypothesis was done by first taking the mean (over time) of the bilateral skin conductance difference for each subject within each period (pre-LARA period, LARA1, and LARA2 periods), and conducting a one-way repeated measures ANOVA with Greenhouse-Geisser correction on these three groups of between-subjects observations. Normality of distribution was tested by the Shapiro-Wilk test, and the Friedman-test (non-parameteric repeated measures ANOVA) was employed for non-normal data. This was followed up with pairwise group comparisons using multiple comparison correction by Tukey or Dunn’s method according to the distribution being normal or non-normal respectively. This analysis was done for both the signed bilateral difference (allowing both positive and negative differences) and its absolute value.

These data representations and statistical analyses were also conducted separately for the subgroup of subjects which had a maximum desire (score = 10) of winning the price in the LARA2 task. In the end, the recordings were visually inspected for any particular cases possibly demonstrating asymmetry.

## Results

**FIGURE 1:**
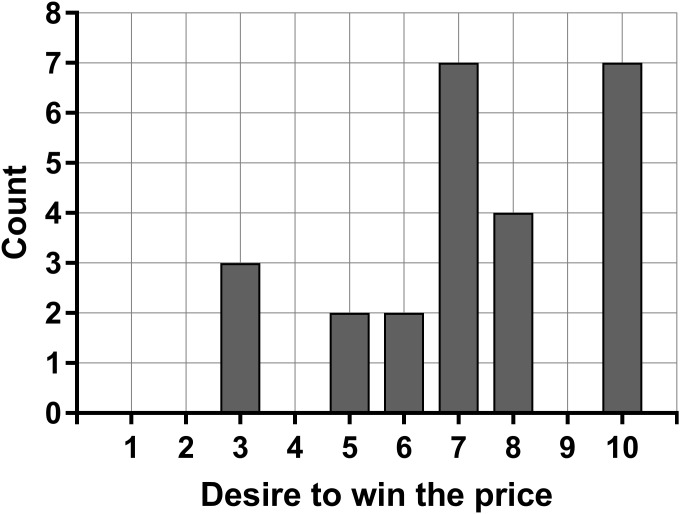
Distribution in the desire to win the price (1–10) between all the participants. Seven participants (28%) rated their desire to win the price at maximum.

The participants rated their desire to win the price with a range from 3 to 10 (on a scale from 1 to 10), with modes at 7 and 10, and a mean of 7.3 (SD = 2.3). Only 3 of the 25 participants rated below 5. The distribution in winning desire among the participants is presented in Figure 1. There was no significant association between winning desire and the bilateral skin conductance difference during the LARA 2 task, neither for the signed difference (rho = 0.15; *p* = 0.47) nor the absolute difference (rho = -0.04; *p* = 0.86) based on the Spearman rank-order correlations.

**FIGURE 2:**
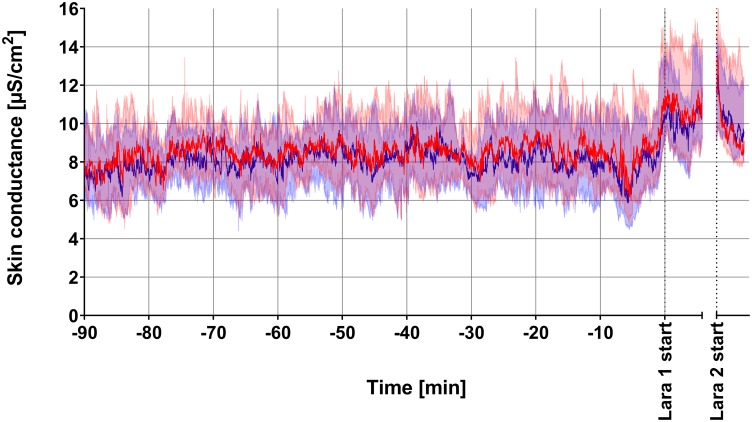
Median and 95% confidence bands for skin conductance at the dominant (red) and non-dominant (blue) hand from the 25 subjects. The purple area (from red and blue semi-transparent overlay) shows the overlap between the two confidence bands.

**FIGURE 3:**
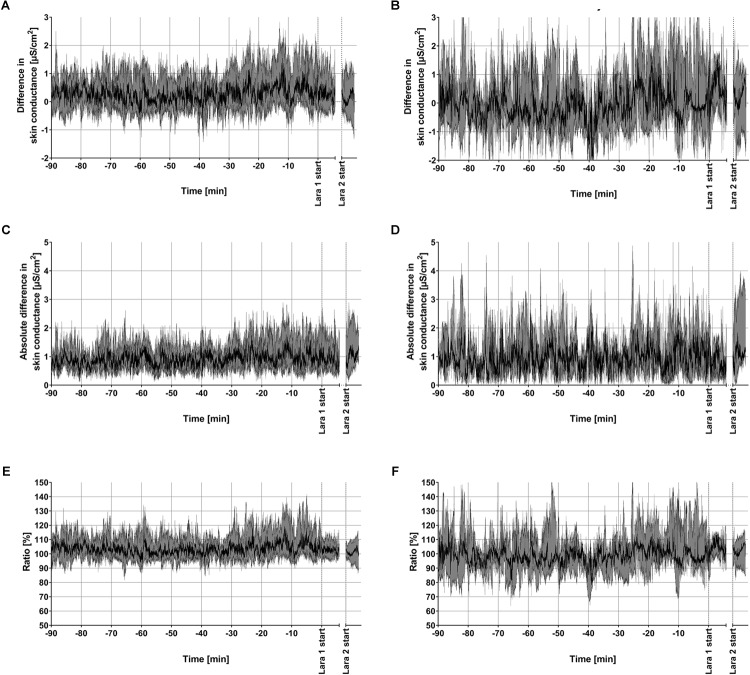
Assessment of bilateral difference in EDA during the experiments, quantified by subtracting the SC of the non-dominant hand from the SC of the dominant hand **(A,B)**, the absolute value of the difference between the sides **(C,D)**, and the ratio between the SC of the dominant and non-dominant hand **(E,F)**. The median and the 95% confidence bands of all 25 subjects are shown in **(A,C,E)**, while **(B,D,F)** present the same statistics based on a subgroup of only the subjects having a maximum winning desire in the LARA test (*n* = 7).

Over the whole recording period, there was a slightly higher skin conductance at the dominant hand, based on individual comparison of means of both sides. The median of the ratio between the mean skin conductance of the dominant and non-dominant hand was 104%, with the 25/75% quartiles at 97 and 114%, respectively. A graphical summary of all recordings is presented in Figure 2, showing the medians and 95% confidence bands of the skin conductance at the dominant and non-dominant hand. The increase in skin conductance from the stress tasks in Phase 2 can be clearly seen from the figure, but the magnitude of bilateral difference during Phase 2 seems to be the same as during Phase 1. For quantitative assessment of bilateral asymmetry, the dominant vs. non-dominant difference, absolute value of difference and ratio are presented in Figure 3. Inspecting plots (A,C,E), these bilateral differences look small throughout the recording and unchanged during the two LARA stress tasks. The same presentation for only the participants having maximum desire to win the price (score = 10, *n* = 7) is given in plots (B,D,F), showing similar results, but with wider confidence bands. Figure 3D indicates that a few recordings contain an increase in the absolute value of bilateral difference in the transition from LARA1 to LARA2, in line with the hypothesis. This was due to two recordings, which are plotted in Figure 5A,B.

**FIGURE 4:**
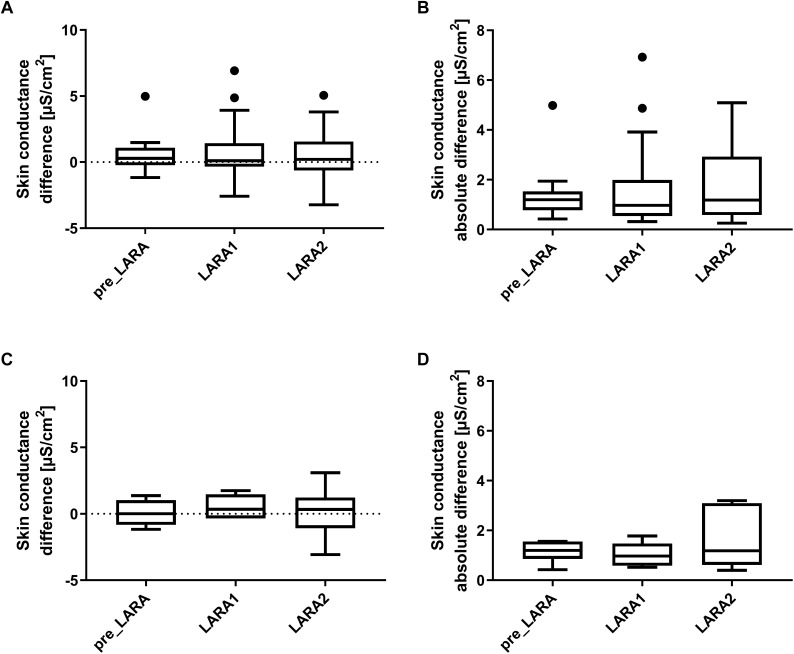
The bilateral skin conductance difference within three phases: the pre-LARA period, as well as during the first and during second LARA test, calculated as **(A)** the mean difference between the dominant and non-dominant hand for all subjects, **(B)** the mean absolute difference between the hands for all subjects, **(C)** the mean difference between the dominant and non-dominant hand for the subgroup with maximum winner wish (*n* = 7), and **(D)** the mean absolute difference between the hands for the subgroup with maximum winner wish. None of the phases were significantly different from another (repeated measures ANOVA with Tukey’s multiple comparison test). The black dots are individual values below the 25% percentile minus 1.5 interquartile range or above the 75% percentile plus 1.5 interquartile range, according to the Tukey method.

Statistically, there was no significant change in the bilateral difference going from the pre-LARA period to the LARA tests, neither for the signed difference (dominant hand minus non-dominant hand) nor the absolute value of the difference, as shown in Table 2 and Figure 4A,B, respectively. This was also the case when analyzing the subgroup with maximum winning desire in the same way (although with lower statistical power having only *n* = 7), shown in Figure 4C,D.

**Table 2:** Descriptive and inferential statistics for the bilateral EDA difference in skin conductance over the three recording periods.

Case	Pre-LARA	LARA1	LARA2	Between groups	Pre-LARA vs. LARA 1	Pre-LARA vs. LARA2	LARA 1 vs. LARA 2	
Statistics	median (95% CI)	median (95% CI)	median (95% CI)	ANOVA^∗^ *p*-value, Friedman statistic	*p*-value, Rank sum difference	*p*-value, Rank sum difference	*p*-value, Rank sum difference	*df*
Signed difference, all participants (*n* = 25) [μS/cm^2^]	*0.28 (-0.09, 0.98)*	*0.11 (-0.35, 1.36)*	*0.21 (-0.58, 1.23)*	*0.05, 6*	*1.0, 0*	*0.10, 15*	*0.10, 15*	*24*
Absolute difference, all participants (*n* = 25) [μS/cm^2^]	*1.20 (0.86, 1.39)*	*0.97 (0.58, 1.51)*	*1.18 (0.62, 2.06)*	*0.85, 0.32*	*1.0, -2*	*1.0, 2*	*1.0, 4*	*24*
Statistics	mean (95% CI)	mean (95% CI)	mean (95% CI)	ANOVA# *p*-value, *F*-value	*p*-value, *q*-statistic	*p*-value, *q*-statistic	*p*-value, *q*-statistic	*df*
Signed difference, selected participants (*n* = 7) [μS/cm^2^]	*0.044 (-0.66, 0.75)*	*0.47 (-0.18, 1.13)*	*0.07 (-1.36, 1.49)*	*0.61, 0.39*	*0.48, 1.73*	*1.00, 0.04*	*0.74, 1.08*	*6*
Absolute difference, selected participants (*n* = 7) [μS/cm^2^]	*1.11 (0.80, 1.42)*	*0.99 (0.63, 1.34)*	*1.48 (0.61, 2.35)*	*0.39, 0.91*	*0.81, 0.90*	*0.74, 1.08*	*0.48, 1.74*	*6*


*Based on testing for normal distribution (Shapiro-Wilk test), parameteric# or non-parameteric^∗^ repeated measures ANOVA was conducted accordingly. Greenhouse-Geisser correction was employed for the parametric ANOVA. For the multiple comparison between the periods, Tukey’s test was employed following the parametric ANOVA, and Dunn’s test was employed following the non-parametric ANOVA. 95% confidence intervals (CI) are provided for the bilateral differences, calculated based on the bootstrap method for the medians and based on the standard error for the means.*

**FIGURE 5:**
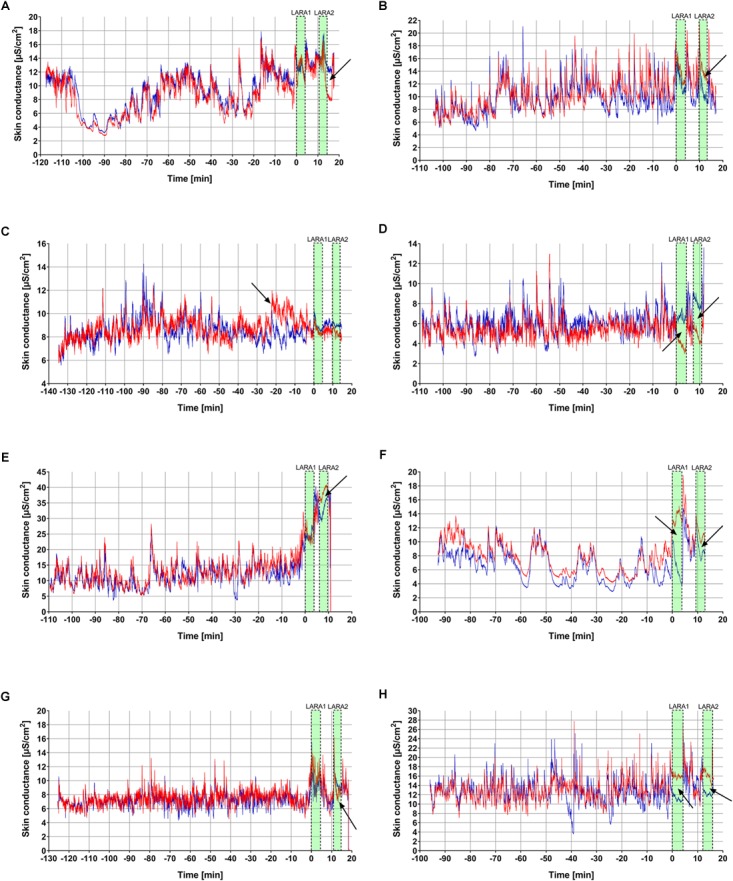
Eight individual recordings **(A–H)** from the study manually picked, suggestive of bilateral EDA asymmetry, where the interesting points are indicated with arrows. Red, dominant hand; blue, non-dominant hand. The duration of the LARA tests are marked with the green boxes.

Figure 5 shows a collection of recordings containing events of possible asymmetry based on visual inspection by the authors, showing cases of both the dominant and non-dominant hand lying higher than the other, mostly occurring during the LARA tests, but also within the pre-LARA periods (Figure 5C).

## Discussion

Based on their observations of human subjects displaying large asymmetries between skin conductance measurements in the left versus the right side of the body, Picard et al. (2016) hypothesized different cortical and subcortical structures to be involved in the processing of various facets or types of emotional states, with the location of these structures accounting for a diversity of lateral biases in prompting EDA reactivity.

In present laboratory-based study, we investigated bilateral effects of skin conductance in a high-stake threat-related scenario in 25 healthy subjects during high and low-stake stress tasks, and found a small bilateral difference (median <0.5 μS/cm^2^) where the 95% confidence intervals overlapped with zero for all conditions, and with no statistically significant effect between them. Consequently, in summary, we were not able to replicate the findings reported in the retrospective analysis by Picard and colleagues. However, among the 25 recordings, there were cases suggestive of bilateral asymmetry at some time during the recording (Figure 5). Nevertheless, among these eight participants, only two displayed EDA patterns conform with the theory that the EDA at the dominant hand increases compared to the non-dominant hand after transition from LARA1 to LARA2 (Figure 5B,E). These eight examples were picked by visual inspection by the authors, but the rest of the data is available and open to interpretation. The interpretation of the observed occurrence or lack of bilateral asymmetry is however a complicated task. Hypothetically, there are many possible factors contributing to the possible bilateral asymmetry in measured skin conductance:

(1) Bilateral difference in the nervous stimulation of the sweat glands, governed by e.g., right-hemispheric amygdala activation.
(2) Peripheral neuropathies causing asymmetric nervous stimulation of the sweat glands.
(3) Difference in the density of active sweat glands below the measuring electrode.
(4) Difference in the electrode to skin contact (effective electrode area).
(5) Movements in electrode positioning at skin areas with spatial differences in sweat gland density [e.g., different areas of the palm as shown in Freedman et al. (1994)].
(6) Changes in skin properties due to electrodes (i.e., occlusion, electrolyte effects or skin irritation) or instrumentation [polarization of skin or electro-osmosis when using DC measurement (Pabst et al., 2017a,b)].
(7) Instrumentational error (i.e., calibration difference between devices or device channels)
(8) Differences in skin temperature between hands

Among these, point 1 is in line with the multiple arousal theory, but this asymmetry should in this case be present in more than a few subjects unless the protocol was inadequate in producing conditions necessary to elicit this feature. Point 2 is unlikely for the participants in this study (diabetes and nerve injury were exclusion criteria). Because the study was conducted in a controlled environment with fixed placement of adhesive gel electrodes particularly selected for stable and sensitive skin conductance measurement (Tronstad et al., 2010), and using AC voltage excitation for measurement, points 5–6 were also unlikely in this study. Sliding of electrodes along the skin could cause changes in the measurement and spurious bilateral differences (due to the spatial distribution in sweat gland density and changes to the electrode-skin contact), but the electrodes used in this study did not allow sliding to occur. Some of these possible causes (nr 3, 6, and 7) should mainly produce constant differences in bilateral measurement, or differences evolving gradually over time, not transient differences as seen in e.g., Figure 5C. On average, the skin conductance in the present study was slightly higher at the dominant hand (0.34 μS/cm^2^), which is in agreement with previous studies (Román et al., 1989). Skin temperature may in general modulate EDA, and difference in external temperature influence between both hands could possibly cause bilateral EDA measurement unrelated to arousal, as demonstrated in Deltombe et al. (1997) for the sympathetic skin response upon large changes in skin temperature. We believe that this effect is very unlikely in our study, as equal conditions were present for both hands, and any changes in environmental temperature would have equal effect on the recording from both hands, as they were measured simultaneously. We cannot exclude point 4, that there were periods of changes in the electrode contact with the skin, such as loosening of adhesion, pressure on electrodes or pulling on wires during the experiment.

The scope and extent of our study is not suitable for a conclusive judgment on a possible multiple arousal theory. Given the early stage of the research on this topic and the previously stated need for more and systematic hypothesis-driven research on this question (Mendes, 2016; Richter, 2016; Sabatinelli, 2016), we propose in the following a range of potential explanatory approaches and suggestions for research designs that will hopefully stimulate further research on a possible multiple arousal theory.

Following the assumption that the multiple arousal theory produces bilaterally asymmetrical EDA effects in high-stake situations, an obviously possible reason for the absence of these effects in the majority of our participants might be that the “threat-level” induced by the loss-aversion paradigm was not intense enough. While only future research with alternative designs can describe the association between experienced threat intensity, perceived threat probability and possibly resulting bilateral EDA asymmetries, the paradigm that we applied most likely had a high ecological validity and a higher objective price “on stake” than many common computer-simulated loss-aversion paradigms which nevertheless have shown stable behavioral effects applying virtual currencies without any real-life effects (e.g., De Martino et al., 2006; Sütterlin et al., 2011). In addition, the desire to obtain the price was generally rated as high and did not seem to be related to EDA levels. On the other hand, the scenarios described by Picard et al. (2016) referred to high-stake threats in social situations. It has been previously shown that social situations have the potential to elicit particularly high intensive threat experiences (e.g., Kirschbaum et al., 1993; Eisenberger, 2012). Our paradigm however, was not socially salient and future research should therefore consider to include socially elicited stressful situations, such as the highly standardized and well-established Trier Social Stress Test (Kirschbaum et al., 1993). In Kasos et al. (2018), significant lateralization of EDA was found due to emotions of fear and sadness induced by music. In addition to the difference in the test paradigm and emotional states compared to our study, Kasos et al. (2018) analyzed single EDA responses to short specific stimuli (7s music excerpts) while our study compared EDA levels over longer time durations, similar to the Picard et al. (2016) study. Whether or not *phasic* EDA parameters would show bilateral differences with respect to theory was not planned for nor explored in this study. The dominant and non-dominant hand dichotomy was used for this bilateral EDA investigation, but the difference in results would be very small if the left-right hand dichotomy was used instead, as only one of the 25 subjects was left-handed. In addition, our analysis on the *absolute value* of bilateral difference is independent on the left/right hand assignment, where neither any statistically significant difference between periods was found.

### Multidimensionality of Emotional States

Importantly, future research should be conceptually clear about the emotional facets under investigation. Examples of threat-related «high-stake» situations in which the relevant data were collected by Picard et al. (2016) included a visit in an amusement park (that was first announced, and then at risk of being called off) and a highly relevant investor meeting. We agree that both of these scenarios are in line with the notion of “high-stake” and “threat,” where the term “threat” implies an element of uncertainty, a risk, the probability of a negative event. A “high-stake threat” would therefore describe an anticipated situation with the probability of a very relevant negative experience. In contrast, the observations of a long-lasting asymmetry during the experienced death of a family member does not meet the probabilistic criterion of a present uncertainty/risk/threat. While all of the reported scenarios seem to share the characteristic of high levels of “arousal” [but see Richter (2016) on the problem of the definition of arousal], they nevertheless include both high-risk and no-risk situations, positive and negative emotional valence, approach- and avoidance-related motivational patterns such as fear, anxiety, pleasant anticipation, happiness, and sorrow. From a psychological perspective, the scenarios interpreted by Picard et al. (2016) can be therefore considered to be highly heterogeneous. In contrast, our present study made use of the single loss-aversion paradigm in the attempt to generate a high-stake situation with a threat. We therefore only covered parts of the experiential facets that were accounted for in Picard’s retrospective analysis. We conclude that in the future studies, the situational conditions in which bilateral asymmetries may occur need to be clearly defined, differentiated, and made subject to controlled conditions to rule out coincidental findings and narrow down the underlying psychophysiological associations of the multiple arousal theory.

### Need for Systematic and Differentiated Research Approaches

Emotions can be independent and multiple (Diener and Emmons, 1984). Depending on the time perspective under consideration, but generally at all given moments, more than one specific emotion can be present, including positive and negative emotions. This is acknowledged by the fact that established self-assessment tools assess positive and negative affect on separate scales (e.g., PANAS; Watson et al., 1988). Correlation between affective states depends on the exact type of combination of affect and are sensitive to the time span that is considered, with shorter time windows typically related to stronger negative correlations between the intensity of distinct but co-existing affective states while longer time windows allow for more diversity (for example sorrow and happiness; or sadness and pride). The extensive overview by Kreibig (2010) displays impressively how even changes in subtle facets of affective states can cause an impressive variety of peripheral physiological responses that per today still do not allow for reliable conclusions on actually experienced mental emotional states. The association between psychophysiological arousal patterns and subjective experience is still very little understood and requires an enormous amount of differentiation. Thus, a systematic investigation of the multiple arousal theory via a range of elicited emotional valences and intensities is required to identify the underlying principles between central nervous and peripheral physiological correlates of subjective experiences such as possibly “threat” or “high salience.” A systematic variation of stake (high- vs. low-stake conditions), emotional valence (positive vs. negative), motivational direction (approach/avoid) and arousal levels (high vs. low) with concurrent monitoring of EDA laterality would therefore contribute to a deeper understanding.

### Assess All Data Levels

The multiple arousal theory makes assumptions on central nervous activity causing peripheral physiological effects. To fully corroborate potential reverse interpretations of CNS correlates of emotional arousal, EDA recordings should be accompanied with simultaneous assessment of CNS-activation (e.g., fMRI) and complementary assessment of the participant’s subjective experience. By including assessment in the psychological domain via self-report measures, motivational directions could be accounted for. Negative valence can, for example, be elicited by the same situational context and in different individuals be associated with approach (aggression, anger) and/or avoidance motivation (fear, anxiety), while both systems trigger very distinct neuronal activation patterns on multiple cortical and subcortical sites and consequences for hemispheric lateralization (e.g., Carver and White, 1994; Hewig et al., 2006; Rutherford and Lindell, 2011). Based on this well-established research on the associations between CNS activation patterns within and across hemispheres with motivational directions of experienced emotional states, it seems to be highly recommended to include self-report data in future studies that could shed a light on whether a high-stake situation is more related to the positive excitement about the chance of winning or the anxiety of losing.

The possibility of obtaining new information by analyzing signals of EDA from different skin sites is a rather unexplored area of research, and is becoming more and more relevant as sensors are being miniaturized with wireless integration allowing ambulatory recording of EDA at multiple skin sites. Not only the traditional recording sites of the palms and fingers provide EDA, but also at numerous other locations on the body (Van Dooren et al., 2012). If bilateral differences in EDA can discriminate psychological stress states, multi-site EDA sensor systems may be even more attractive.

## Conclusion

In the present study, a standardized laboratory setup in which a possible loss of an attractive object created a threat-related high-stake situation, did not lead to consistent observation of bilateral asymmetries in EDA. Our laboratory-based study did not replicate the retrospective findings reported by Picard et al. (2016) and the present results do therefore not provide support for the previously proposed multiple arousal theory. The current study’s scope and extent, however, is not suitable for a conclusive judgment on a possible multiple arousal theory. Future research needs to systematically differentiate between the various domains of emotional experience (e.g., valence, intensity, motivation) in well-controlled standardized studies and incorporate more comprehensive methodological approaches on the peripheral- and central physiological as well as the subjective psychological data levels.

## Ethics Statement

The study has been approved for implementation by the Regional Committees for Medical and Health Research Ethics (REK; ref. 2016/333). This study was carried out in accordance with the recommendations of the Regional Committees for Medical and Health Research Ethics (REK Sør-Øst) with written informed consent from all subjects. All subjects gave written informed consent in accordance with the Declaration of Helsinki. The study was evaluated by REK Sør-Øst (ref. 2016/333), considered not to be requiring further assessment by the committee (outside of the Health Research Act §2), and approved for implementation without changes or additional scrutinization.

## Author Contributions

AB: planning of the study, recruiting volunteers, conducting experiments, data analysis, and contribution in writing of the manuscript. FP and SM: planning of the study, recruiting volunteers, conducting experiments, and contribution in writing of the manuscript. CT: planning of the study, providing equipment, supervision, data analysis and statistics, and major contribution in writing of the manuscript. HK: planning of the study, providing equipment, supervision, and contribution in writing of the manuscript. SW: planning of the study, interpretation of results, and contribution in writing the manuscript. OP: interpretation of results, data analysis, and contribution in writing the manuscript. SS: planning of the study, interpretation of results, and major contribution in writing of the manuscript.

## Conflict of Interest Statement

The authors declare that the research was conducted in the absence of any commercial or financial relationships that could be construed as a potential conflict of interest.
